# The AI doctor will see you now: public perspectives on artificial intelligence in healthcare

**DOI:** 10.1093/bjrai/ubaf003

**Published:** 2025-02-20

**Authors:** Carolyn Horst, Muhammad Aniq, Alice Taylor-Gee, Jennifer Wong, Vicky Goh

**Affiliations:** School of Biomedical Engineering & Imaging Sciences, King’s College London, London SE1 7EU, United Kingdom; Guy’s & St Thomas’ Hospitals NHS Foundation Trust, Department of Radiology, St Thomas' Hospital, London SE1 7EH, United Kingdom; Guy’s & St Thomas’ Hospitals NHS Foundation Trust, Department of Radiology, St Thomas' Hospital, London SE1 7EH, United Kingdom; School of Biomedical Engineering & Imaging Sciences, King’s College London, London SE1 7EU, United Kingdom; Science Gallery London, King’s College London, London SE1 9GU, United Kingdom; School of Biomedical Engineering & Imaging Sciences, King’s College London, London SE1 7EU, United Kingdom; Guy’s & St Thomas’ Hospitals NHS Foundation Trust, Department of Radiology, St Thomas' Hospital, London SE1 7EH, United Kingdom

**Keywords:** artificial intelligence, radiology, patient and public involvement, quantitative survey

## Abstract

**Objectives:**

The use of artificial intelligence (AI) in healthcare is a growing field of research and clinical application. The views of the general public, that is, current and future healthcare users, need to be surveyed and interpreted so that researchers and the public have a shared understanding of the appropriate use of AI. Currently, there are only limited data on the public’s views. The aim of this study is to understand the public’s perspective on the use of AI in healthcare.

**Methods:**

An anonymous, quantitative questionnaire was administered as part of a public exhibition on AI. The questionnaire contained 8 questions based on previously validated subject areas designed to assess respondents’ views on the use of AI in healthcare. Brief demographic data were also collected.

**Results:**

The population surveyed was more diverse and younger than the general UK population (64% White, 45% aged 18-29). Respondents were largely comfortable with the application of AI in healthcare: 80% felt positively about its use, 56% thought it would be safe. Seventy-one percent did not feel that it would replace doctors, and most would not be happy for AI to make decisions without considering their feelings.

**Conclusions:**

Our study shows that the subset of the general public we surveyed, largely comprised of young, likely future healthcare users, is comfortable with the use of AI in healthcare, but does not see it as a replacement for doctors.

**Advances in knowledge:**

This article highlights views from a subset of the general public on the use of AI in healthcare, which is largely under researched.

## Introduction

Artificial intelligence (AI) tools have the potential to be used in multiple and complex ways within healthcare.^1^^,^^2^ In the last 10 years, increasing technological maturity has meant that these tools have been applied to multiple clinical scenarios, largely in the context of research and application development.^1^^,^^3^ However, real-world clinical deployment is happening, and rapidly expanding, with over 200 FDA-approved products for radiology alone on the market and many more in the pipeline.^4^ These advances have been aided by the advent of large clinical datasets and have the potential to meet a significant clinical need, not least in the NHS, where efficiency is at a premium in a cash-strapped healthcare landscape.

In general, it appears physicians, and particularly radiologists, are comfortable with the application of AI in the clinical sphere: it is seen not as an existential threat, but instead as a tool to improve their performance.^5–9^ There has also been some research into patients’ preferences on the use of AI in their care, which demonstrates some ambivalence about its use and greater acceptance with increased understanding of its role.^10–12^ What has largely been underexplored is the general public’s views on the use of AI in healthcare, with only a limited amount of academic research published in this area.^10^^,^^13^ The NHS did administer a study (*n* = 1031) on AI in healthcare in 2022, using specific scenarios of potential AI applications, however, more general attitudes towards AI were not surveyed.^14^

The perspective of the general public is important for successful deployment of AI in healthcare, not least in the United Kingdom, where taxation funds the healthcare system. And whilst patient views are integral to this process, so are the views of *future* healthcare users, a group likely to skew towards a younger demographic than current patients, and who therefore may have different ideas about AI and its application. The objective of this study was to survey a subset of the general public to understand their perspectives on the use of AI in healthcare.

## Methods

### Questionnaire development

**Figure 1.**
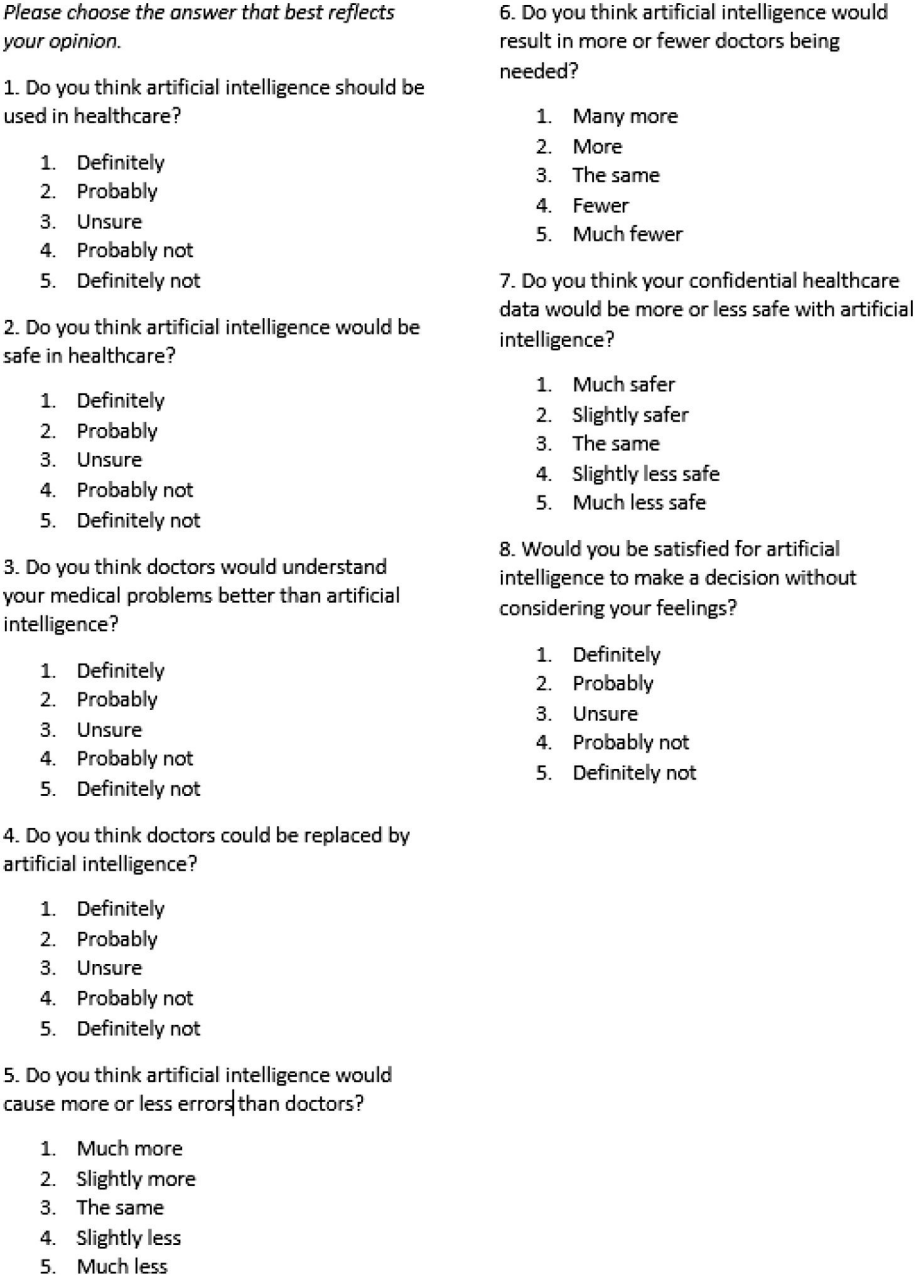
Survey questions.

The questionnaire was approved by the King’s College London (KCL) College Research Ethics Committee (CREC). The questionnaire was based on a previously validated set of subject-specific topics using a Likert-type scale.^11^ These questions aimed to assess views on the safety and utility of AI in healthcare, as well as whether AI was felt to be comparable to the role of a doctor. The survey contained 8 questions as well as demographic data including age, ethnicity, gender, and the first 3 digits of their postcode (see Figure 1 for an illustration of the questions).

### Questionnaire administration and population

This questionnaire was administered as part of an exhibition at Science Gallery London in the United Kingdom. The exhibition, entitled *AI: Who’s Looking After Me?*, was open to the general public from June 2023 to January 2024 and attracted over 30 000 visitors. Visitors to the exhibition were invited to complete the questionnaire anonymously. Consent and administration of the survey were conducted via an unassisted tablet computer displaying a web form. No identifiable information was collected and there was no opportunity to provide identifiable information within the questionnaire.

### Data analysis

Data were analysed for trends including correlation of comfort or discomfort with AI and demographic information, including age and sex. A limited geographical analysis was performed based on limited postcode information.

## Results

2070 responses were collected, of which 55% of respondents identified as female, 45% were between 18 and 29 years of age, and 64% identified as White (see Table 1 for full demographic data). For context, 82% of people in the United Kingdom identify as White. Four hundred and thirty-four (21%) responses to the postcode demographic question were invalid, but based on a limited analysis of the remaining respondents, participants represented a range of locations in the United Kingdom, including the most and least affluent London boroughs, other UK cities including Birmingham, Liverpool, and Manchester, as well as rural areas. Over half (1167) of respondents were resident in a greater London postcode (Table 2).

**Table 1.** Demographic details of survey respondents.

Age	
18-29	931
30-49	750
50-69	307
70-89	51
90+	31
Gender	
Female	1137
Male	777
Neither female nor male	62
Prefer not to say	94
Ethnicity	
Asian or Asian British	358
Black, African, Caribbean or Black British	75
Mixed or Multiple ethnic groups	121
Other ethnic group	89
Prefer not to say	112
White	1315
Total	2070

**Table 2.** Responses to the first 3 digits of postcode.

Total responses	2070
Valid responses	1636 (79%)
London postcode	1167 (71%)
Non-London postcode	469 (29%)

**Figure 2.**
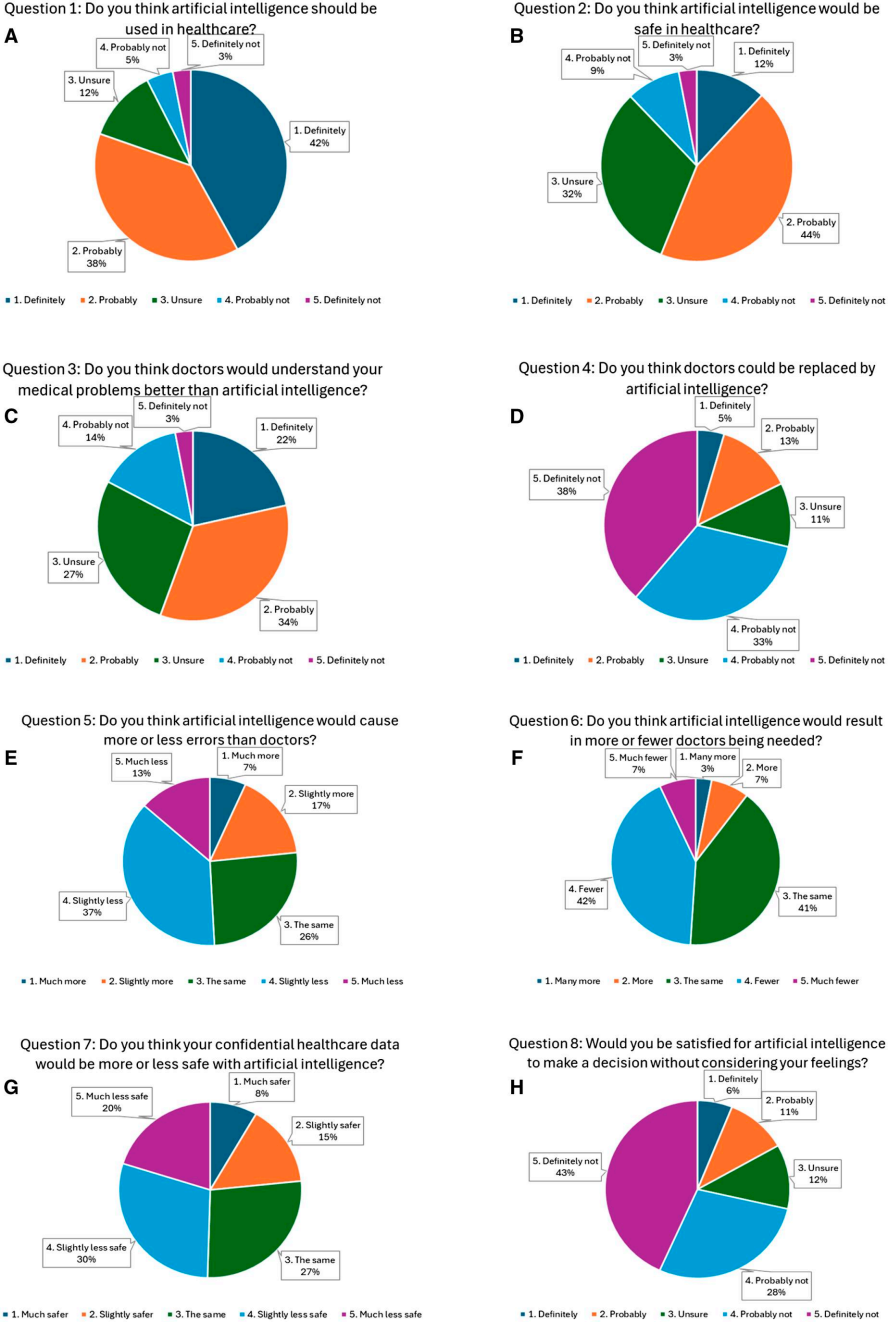
(A-H) Responses to the 8 different subject-specific questions in the questionnaire.

Figure 2 is a graphical representation of the answers to each of the 8 research questions. Overall, 80% responded that AI should definitely or probably be used in healthcare. Fifty-six percent felt that it would be safe, with 32% saying they were unsure. Over 70% did not think doctors would be replaced by AI and half felt AI would make fewer mistakes than doctors. People were largely unhappy with the idea of AI programmes making decisions without considering their feelings (71%) and a majority believed doctors are better placed to understand medical problems than AI (56%).

Subgroup analyses demonstrated that older age groups (defined as 50 years and above) tended to view AI in healthcare as safer (62% vs 55% in under-50s), but otherwise did not differ widely from younger age groups in their responses (see Table 3). Men were more comfortable with AI implementation in healthcare (88% vs 77% for women), and were more likely to think it safe (65% vs 52%) and that it would cause fewer errors (59% vs 46%). Ethnicity did not appear to correlate with positive or negative responses.

**Table 3.** Survey answers by demographic subgroup.

	Age		Gender				Ethnicity					
	Age ≥ 50	Age < 50	Male	Female	Neither female nor male	Prefer not to say	Asian or Asian British	Black, African, Caribbean or Black British	Mixed or Multiple Ethnic Groups	Other Ethnic Group	Prefer Not to Say	White
**Totals**	389	1681	777	1137	62	94	358	75	121	89	112	1315
**Q1: Do you think artificial intelligence should be used in healthcare?**												
1. Definitely	190	679	427	393	19	30	147	27	46	38	35	576
2. Probably	138	657	249	487	28	31	143	33	51	29	36	503
3. Unsure	40	211	71	157	5	18	42	9	12	11	23	154
4. Probably Not	7	85	15	67	4	6	16	1	10	6	7	52
5. Definitely Not	14	49	15	33	6	9	10	5	2	5	11	30
**Q2: Do you think artificial intelligence would be safe in healthcare?**												
1. Definitely	59	186	139	89	8	9	52	8	15	14	12	144
2. Probably	182	734	365	501	23	27	159	38	45	35	31	608
3. Unsure	117	541	209	396	16	37	98	20	48	21	47	424
4. Probably Not	18	170	48	119	8	13	41	4	10	12	14	107
5. Definitely Not	13	50	16	32	7	8	8	5	3	7	8	32
**Q3: Do you think doctors would understand your medical problems better than artificial intelligence?**												
1. Definitely	92	353	165	249	8	23	107	21	19	21	31	246
2. Probably	129	576	258	396	24	27	125	22	41	29	31	457
3. Unsure	104	458	208	311	17	26	82	17	37	28	28	370
4. Probably Not	47	249	117	157	9	13	39	12	21	7	14	203
5. Definitely Not	17	45	29	24	4	5	5	3	3	4	8	39
**Q4: Do you think doctors could be replaced by artificial intelligence?**												
1. Definitely	25	71	64	23	3	6	19	10	4	5	5	53
2. Probably	55	217	114	138	6	14	61	7	17	17	8	162
3. Unsure	50	175	95	106	9	15	42	9	13	11	22	128
4. Probably Not	129	549	273	367	17	21	128	20	41	16	28	445
5. Definitely Not	130	669	231	503	27	38	108	29	46	40	49	527
**Q5: Do you think artificial intelligence would cause more or less errors than doctors?**												
1. Much More	32	106	40	77	9	12	32	10	5	4	13	74
2. Slightly More	46	299	112	208	10	15	79	9	21	13	15	208
3. The Same	107	428	165	324	17	29	78	17	34	32	41	333
4. Slightly Less	149	624	308	422	15	28	129	25	43	28	28	520
5. Much Less	55	224	152	106	11	10	40	14	18	12	15	180
**Q6: Do you think artificial intelligence would result in more or fewer doctors being needed?**												
1. Many More	15	48	19	29	3	12	16	5	4	5	8	25
2. More	26	125	48	90	4	9	38	7	10	3	9	84
3. The Same	181	661	309	478	21	34	120	27	53	38	44	560
4. Fewer	140	730	331	478	27	34	157	27	45	35	42	564
5. Much Fewer	27	117	70	62	7	5	27	9	9	8	9	82
**Q7: Do you think your confidential healthcare data would be more or less safe with artificial intelligence?**												
1. Much Safer	40	133	86	73	6	8	30	13	11	9	5	105
2. Slightly Safer	51	260	118	178	7	8	56	14	18	11	11	201
3. The Same	118	443	230	293	16	22	103	14	30	29	29	356
4. Slightly Less Safe	105	503	210	348	17	33	107	23	38	16	34	390
5. Much Less Safe	75	342	133	245	16	23	62	11	24	24	33	263
**Q8: Would you be satisfied for artificial intelligence to make a decision without considering your feelings?**												
1. Definitely	26	100	64	49	3	10	24	7	4	7	9	75
2. Probably	39	183	121	92	4	5	39	6	13	12	6	146
3. Unsure	40	203	108	117	6	12	45	8	16	7	14	153
4. Probably Not	113	470	247	304	14	18	97	16	39	23	22	386
5. Definitely Not	171	725	237	575	35	49	153	38	49	40	61	555
**Totals**	**389**	**1681**	**777**	**1137**	**62**	**94**	**358**	**75**	**121**	**89**	**112**	**1315**

## Discussion

Our study demonstrates that the subset of the general public surveyed in our questionnaire is broadly comfortable with the application of AI tools within healthcare: they largely support its use, and they believe it is likely to be safe and make fewer mistakes than doctors. Interestingly, they do not see AI as a replacement for doctors, and would not be happy for these tools to be used to make decisions without considering their feelings.

This constellation of findings is concurrent with patients’ views as described by Ongena et al. in their initial qualitative research on this subject, as well as subsequent quantitative research amongst patients and the public.^10^^,^^11^ This is also similar to radiologists’ views: recent research indicates that radiologists generally see the role of AI as supplementary and complementary to their role, but not as a threat.^5^^,^^7^^,^^8^

These results underpin 2 important concepts for the successful adoption of AI in healthcare: consent and communication. As key stakeholders in healthcare systems, the views of the public and patients on the use of AI must be understood and, in a general sense, consent obtained. Based on this research, it appears that public see the application of AI in healthcare similarly to patients and physicians, particularly radiologists, and that broadly agree with its use. This shared understanding is likely to support trust in both the AI and the physicians using it. In the future, as AI applications become more complex, explaining to patients how their data will be used and for what purpose is likely to make people more amenable to AI application, not less.^8^

These data also point to a second important concept in the implementation of AI: good communication is paramount for the public, and AI is not expected to play this role. They are understandably unwilling for AI tools to make diagnoses without considering their feelings. Furthermore, AI results often require expert physicians to interpret and synthesize any AI outputs; expecting the public to understand these outputs is unfair. As more medicine is necessarily delivered remotely, and trust in AI increases, the importance of dialogue between the public, patient, and physician should not be neglected.

This study had several limitations: as it was administered in a public space with no supervision, responses were not quality-controlled. Our respondents were largely young (45% between 18 and 29, and a further 36% between 30 and 49), thereby belonging to a group that is more accepting of technology and less likely to be interacting with healthcare on a regular basis. Our population was not perfectly representative of the general UK population regarding ethnicity, with 64% identifying as white, compared with 82% in the general UK population. However, data show that non-White populations are more likely to have poorer health outcomes and therefore have increased use of healthcare services.^15^ Therefore, understanding the perspectives of non-white groups is important and the overrepresentation of these groups in our dataset may be an advantage. Our geographical representation was unsurprisingly skewed by a large cohort of valid responses being from those living in a greater London postcode. This is unsurprising, given that the exhibition was in London, but our data may overrepresent views of those living in a metropolitan area. A final limitation to our study is that the live research environment was implemented within an exhibition on AI, so those completing the survey may have had more interest and/or comfort with the idea of AI than others in the general population. Despite these limitations, our data provide an interesting insight into the public’s feelings around AI in healthcare in the United Kingdom.

In order to maintain the public’s trust in AI and healthcare, we must continue investing in the virtuous circle of providing information and gathering feedback to ensure consent and ongoing good communication. As radiologists, we are at the forefront of the application of AI in the clinical sphere, and we are set to gain in efficiency and accuracy, as long as we understand the tools we are using. Similarly, the future everyday use of AI depends on the patients and the public believing in the tools and our ability to use them well.
